# Maternal morbidity and preterm birth in 22 low- and middle-income countries: a secondary analysis of the WHO Global Survey dataset

**DOI:** 10.1186/1471-2393-14-56

**Published:** 2014-01-31

**Authors:** Joshua P Vogel, Anne CC Lee, João Paulo Souza

**Affiliations:** 1 School of Population Health, Faculty of Medicine, Dentistry and Health Sciences, University of Western Australia, 35 Stirling Highway, Crawley 6009, Australia; 2 UNDP/UNFPA/UNICEF/WHO/World Bank Special Programme of Research, Development and Research Training in Human Reproduction (HRP), Department of Reproductive Health and Research, World Health Organization, Avenue Appia 20, Geneva CH-1211, Switzerland; 3 Department of International Health, Johns Hopkins Bloomberg School of Public Health, Baltimore, MD, USA; 4 Department of Newborn Medicine, Brigham and Women’s Hospital, Boston, MA, USA

**Keywords:** Maternal, Newborn, Morbidity, Spontaneous preterm birth, Provider-initiated preterm birth, Maternal height, Urinary tract infection, Pre-eclampsia, Diabetes, Malaria, HIV

## Abstract

**Background:**

Preterm birth (PTB) (<37weeks) complicates approximately 15 million deliveries annually, 60% occurring in low- and middle-income countries (LMICs). Several maternal morbidities increase the risk of spontaneous (spPTB) and provider-initiated (piPTB) preterm birth, but there is little data from LMICs.

**Method:**

We used the WHO Global Survey to analyze data from 172,461 singleton deliveries in 145 facilities across 22 LMICs. PTB and six maternal morbidities (height <145 cm, malaria, HIV/AIDS, pyelonephritis/UTI, diabetes and pre-eclampsia) were investigated. We described associated characteristics and developed multilevel models for the risk of spPTB/piPTB associated with maternal morbidities. Adverse perinatal outcomes (Apgar <7 at 5 minutes, NICU admission, stillbirth, early neonatal death and low birthweight) were determined.

**Results:**

8.2% of deliveries were PTB; one-quarter of these were piPTB. 14.2% of piPTBs were not medically indicated. Maternal height <145 cm (AOR 1.30, 95% CI 1.10–1.52), pyelonephritis/UTI (AOR 1.16, 95% CI 1.01–1.33), pre-gestational diabetes (AOR 1.41, 95% CI 1.09–1.82) and pre-eclampsia (AOR 1.25, 95% CI 1.05–1.49) increased odds of spPTB, as did malaria in Africa (AOR 1.67, 95%CI 1.32-2.11) but not HIV/AIDS (AOR 1.17, 95% CI 0.79-1.73). Odds of piPTB were higher with maternal height <145 cm (AOR 1.47, 95% CI 1.23-1.77), pre-gestational diabetes (AOR 2.51, 95% CI 1.81-3.47) and pre-eclampsia (AOR 8.17, 95% CI 6.80-9.83).

**Conclusions:**

Maternal height <145 cm, diabetes and pre-eclampsia significantly increased odds of spPTB and piPTB, while pyelonephritis/UTI and malaria increased odds of spPTB only. Strategies to reduce PTB and associated newborn morbidity/mortality in LMICs must prioritize antenatal screening/treatment of these common conditions and reducing non-medically indicated piPTBs where appropriate.

## Background

Preterm birth (birth before 37 weeks gestation) complicates an estimated 15 million deliveries every year, of which 1.1 million infants will die due to preterm birth-related complications [1]. It is the leading cause of death in the first month of life and preterm neonates are at an increased risk for post-neonatal mortality and a wide range of respiratory, infectious, metabolic and nervous system morbidities [2,3]. These risks persist beyond the neonatal period – preterm birth is the second leading cause of death in children under 5 [4] and preterm infants have more illnesses, hospital admissions and educational and behavioral problems in childhood and early adulthood [5-8]. They are also at greater risk of adult chronic diseases, such as hypertension and diabetes [9]. In 2012, the “Born Too Soon: the Global Action Report on Preterm Birth” was released, reporting the first country-level estimates of preterm deliveries and mapping a comprehensive global strategy to address preterm birth [10]. Over 60% of preterm births occur in low- and middle-income countries (LMICs) and the incidence has consistently risen in most countries [1,10]. A worldwide co-operative effort to empower women and improve the availability of family planning and pre-pregnancy and antenatal care may prevent preterm birth, while upscaling simple, low-cost interventions can save the lives of premature infants [10].

Approximately 45-50% of preterm deliveries follow spontaneous onset of labour, 30% follow preterm rupture of membranes and 15-20% of preterm births are provider-initiated [11]. While the etiology of spontaneous preterm birth is heterogeneous and poorly understood, many maternal factors are known to increase risk, such as age (adolescence and advanced age), race, multiple pregnancy, short inter-pregnancy interval, infections, medical conditions, poor nutrition, lifestyle factors, psychological factors and genetic predisposition [12-14]. Provider-initiated preterm birth can be life-saving for both mother and fetus, such as in severe pre-eclampsia, placental abruption or fetal distress. However, some provider-initiated preterm deliveries may not have a strong medical indication or are unintentionally preterm due to gestational age error [10]. Consequently, a significant fraction of the worldwide preterm birth burden may be unnecessary and avoidable.

There is a dearth of large-scale prevalence data from LMICs on the different phenotypes of preterm birth (spontaneous and provider-initiated) and on antenatal maternal morbidities known to contribute to preterm birth, as well as neonatal morbidity and mortality amongst preterm infants. In many resource-constrained settings, conditions that are known to be risk factors for preterm birth (such as urinary tract infections, malaria, undernutrition and hypertensive disorders) are frequently under-diagnosed, under-treated or both. Consequently, the magnitude of the association between maternal morbidities, the risk of preterm birth and neonatal morbidity and mortality in these settings is difficult to establish, despite the availability of proven interventions for these conditions [10].

The Child Health Epidemiology Research Group (CHERG) has conducted a series of analyses exploring the association between maternal risk factors (nutrition, infections and pregnancy morbidity) and intra-uterine growth restriction and preterm birth [3]. As part of that work, we conducted a secondary analysis of facility deliveries in an international WHO dataset to describe the prevalences and relationships between selected maternal morbidities, spontaneous and provider-initiated preterm birth and neonatal morbidity and mortality in preterm infants in 22 LMICs.

## Methods

The WHO Global Survey on Maternal and Perinatal Health (hereafter referred to as WHOGS) is a multi-country, facility-based, cross-sectional survey of maternal and perinatal outcomes following delivery. The methodological details have been previously published [15,16]. In brief, a stratified, multistage sampling design was used to obtain a global sample of countries and health institutions. Countries in WHO regions were grouped according to adult and child mortality and countries were randomly selected (probability proportional to population). Of these, 24 participated in Africa (Angola, Democratic Republic of Congo, Algeria, Kenya, Niger, Nigeria and Uganda), Latin America (Argentina, Brazil, Cuba, Ecuador, Mexico, Nicaragua, Paraguay and Peru) and Asia (Cambodia, China, India, Japan, Nepal, Philippines, Sri Lanka, Thailand and Vietnam). From within the capital city and two randomly selected provinces, seven institutions with over 1,000 births per year and the capacity to perform caesarean sections (CS) were randomly selected. The study was conducted over 2004-2005 (Africa and Latin America) and 2007-2008 (Asia).

All women delivering over 20 weeks gestation in participating institutions during a three-month study period were included. Trained data collectors reviewed and extracted relevant data from medical records to complete the individual data forms on mothers and neonates during admission for delivery until discharge or day 7 postpartum (whichever came first). Maternal or neonatal morbidity or mortality occurring after discharge or day 7 postpartum was not captured. Maternal morbidities available in the WHOGS dataset known to increase the risk of preterm birth were selected as exposure variables, namely: maternal height (as a proxy for maternal malnutrition), malaria, HIV/AIDS (evidence in the hospital records of the woman being HIV positive or having AIDS), pyelonephritis/UTI (history of infection during the pregnancy or delivery), pre-gestational diabetes (women who became pregnant and were known to have diabetes that antedated pregnancy) and pre-eclampsia (blood pressure of 140/90 or greater, or an increase of 30 mm Hg systolic or 15 mm Hg diastolic over baseline values on at least two occasions six or more hours apart, developing after 20 weeks plus proteinuria/albuminuria). These conditions were recorded as binary variables (yes/no), except for maternal height which we stratified into four groups (<145 cm, 145 – 149.9 cm, 150 – 154.9 cm, > = 155 cm) There is no universal definition for low maternal height, however 145 cm and 150 cm are common cutoffs used in anthropometry studies in LMICs ( [17-19] and we reported results for all four height groups (> = 155 cm as reference group). Women were not universally screened for these conditions; they may have been diagnosed at any point during pregnancy. Information on severity, method of diagnosis and management of these morbidities was not recorded in the WHOGS.

We created a primary outcome variable related to preterm birth with three mutually exclusive groups, namely 1) spontaneous preterm birth (spPTB) defined as deliveries occurring before 37 weeks gestation following spontaneous onset of labour, 2) provider-initiated preterm birth (piPTB) defined as delivery before 37 weeks gestation following induction of labour, or no labour and 3) term births (> = 37 weeks gestation, reference group). Medically indicated piPTB were those with one or more documented medical indications, while non-medically indicated piPTB deliveries were recorded as elective or by maternal request only. Those piPTB deliveries with no documented indications (medical or otherwise) were reported as unknown. Perinatal outcomes were: Apgar score <7 at 5 minutes, admission to neonatal intensive care unit (NICU), stillbirth (neonate showing no signs of life at time of delivery), low birthweight (birthweight <2500 g) and early neonatal mortality (ENM) by discharge or day 7. Deaths occurring in the first week of life after discharge (at home or during a postpartum readmission) were not captured.

**Figure 1:**
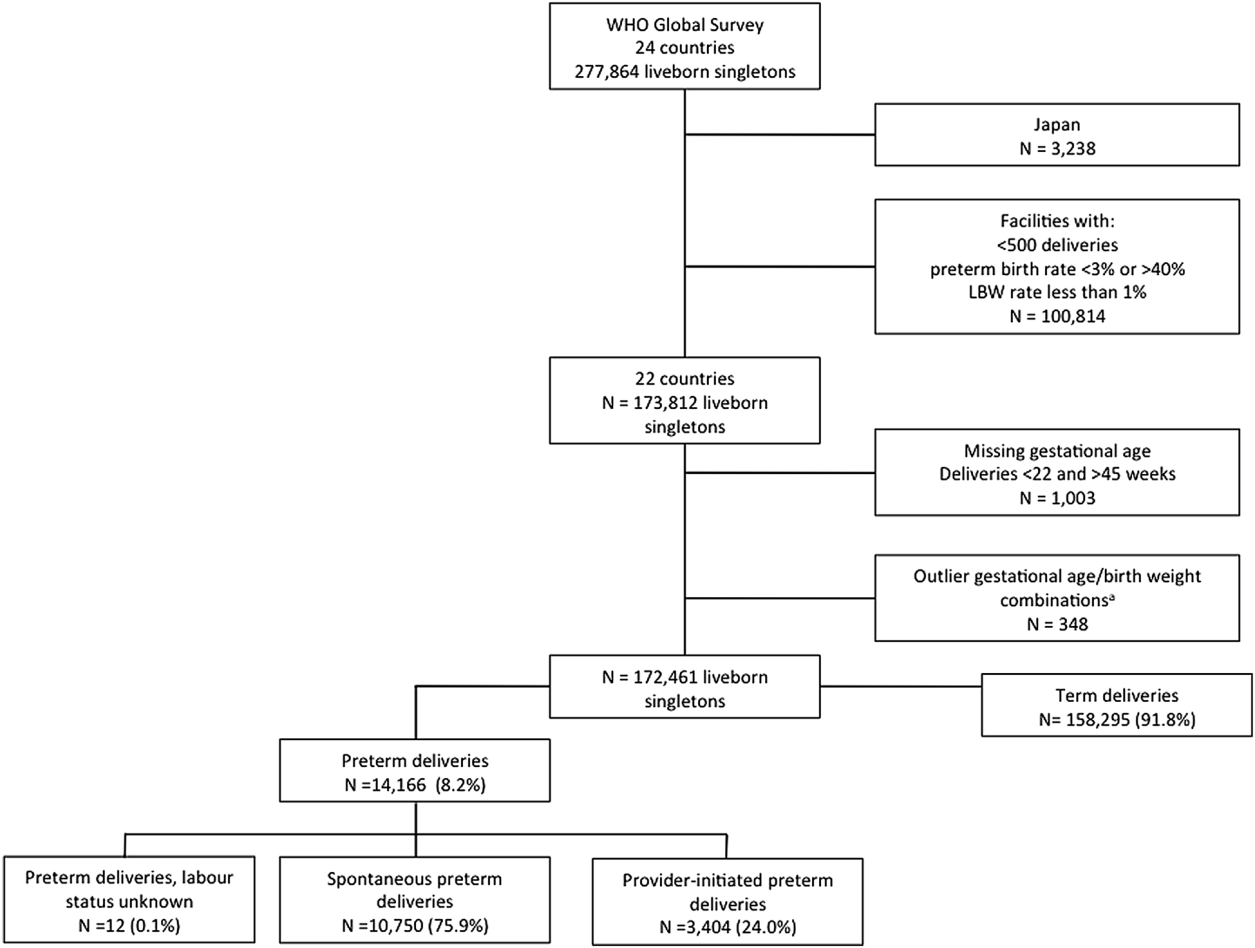
Study flowchart.

The WHOGS captured 290,610 deliveries in 373 institutions across Africa, Latin America and Asia. For this analysis, we used data on liveborn singletons only from the 22 LMICs in the WHOGS dataset, as defined by the Organization for Economic Cooperation and Development (http://www.oecd.org) (Japan was excluded as it is classified as a high-income country). We excluded multiple pregnancies on account of the differences in preterm birth rates between singletons and multiples and controversy surrounding the optimal timing of delivery for multiple births [20,21]. The relationship between maternal morbidities and multiple pregnancies may also differ, potentially leading to over-estimates of effect. We excluded facilities that had less than 500 deliveries, and those with implausible/unrepresentative data or evidence of poor data capture (i.e.: a preterm birth rate of less than 3% or greater than 40%, or a low birthweight rate of less than 1%). Deliveries with a gestational age of 22 to 45 weeks were included, however deliveries with missing gestational age were excluded, as were outlier gestational age/birthweight combinations identified by algorithms developed by Alexander et al. [22] As per these criteria, 172,461 singleton live births from 22 countries available for analysis (Figure 1). All deliveries in Angola were excluded as they did not meet the above data quality criteria.

The prevalences of maternal and neonatal characteristics were reported for spPTB, piPTB and term birth groups. Missing values were reported, however they were excluded from significance testing. Chi-square tests and Student’s t-test tested significance for spPTB vs term and piPTB vs term. We also reported the prevalence of preterm birth groups and maternal morbidities at the country level. Global and regional multi-level logistic regression models (using the GENLINMIXED procedure in SPSS) were used to determine adjusted odds ratios of spPTB and piPTB associated with the maternal morbidities, while accounting for clustering of mothers within facilities. Maternal age, education, marital status, parity, antenatal care, multiple pregnancy and facility (as a random effect) were included as confounders in the multivariate models. The frequency of HIV/AIDS and malaria was extremely low in Latin America and Asia and were thus excluded from these models and the global model. Missing values were excluded from modelling. Perinatal outcomes were reported by gestational age bands (<28, 28 to 31, 32 to 33, 34 - <37 and >37 weeks) and by preterm groups. Ethical clearance from all Ministries of Health of participating countries, WHO Ethics Review Committee and that of each health institution or sub-region were obtained. All analyses were performed using SPSS 20 [23].

## Results

**Figure 2:**
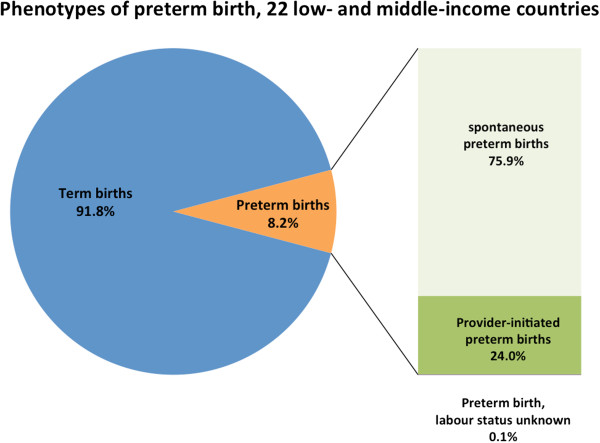
Phenotypes of preterm birth, 22 low- and middle-income countries.

**Figure 3:**
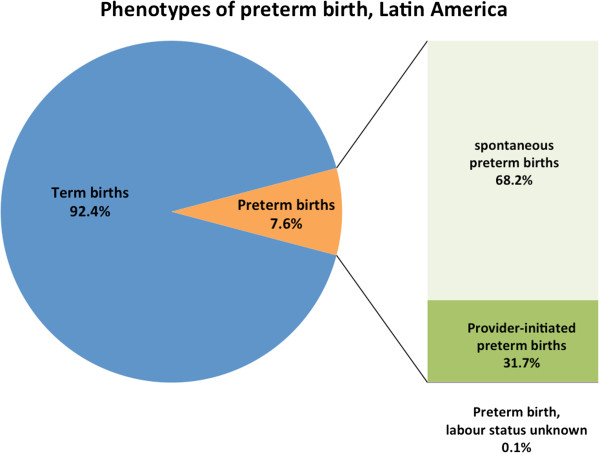
Phenotypes of preterm birth, Latin American countries.

**Figure 4:**
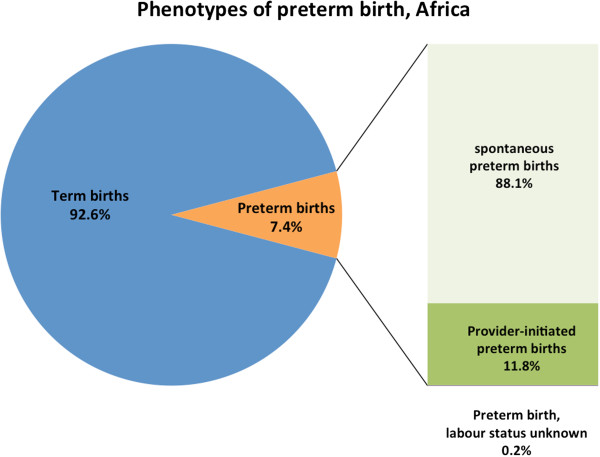
Phenotypes of preterm birth, African countries.

**Figure 5:**
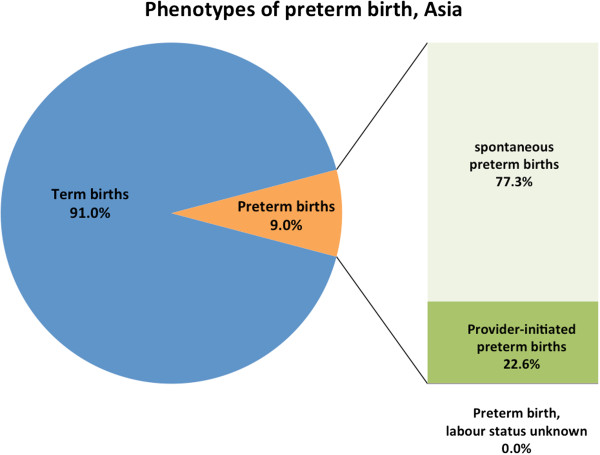
Phenotypes of preterm birth, Asian countries.

Of the 172,461 liveborn singletons in this analysis, 14,155 (8.2%) were preterm (Figure 1). Approximately three-quarters of all preterm births were spontaneous (spPTB) while one-quarter were provider-initiated preterm births (piPTB); this proportion varied by region (Latin America 31.7%, Africa 11.8% and Asia 22.6%) (Table 1, Figures 2, 3, 4, 5). Globally, 85.8% of the provider-initiated preterm deliveries were medically indicated (Latin America 91.1%, Africa 88.7% and Asia 80.1%).

**Table 1:** Prevalence of preterm birth groups, by region

	**Global**	**Latin America**	**Africa**	**Asia**
Number of facilities	122	54	27	64
Number of liveborn singleton deliveries	172,461	64,181	30,544	77,736
Term births (> = 37 weeks)	158,295 (91.8)	59,288 (92.4)	28,284 (92.6)	70,723 (91.0)
All preterm births (< 37 weeks)	14,166 (8.2)	4,893 (7.6)	2,260 (7.4)	7,013 (9.0)
Spontaneous preterm birth (spPTB)^a^	10,750 (75.9)	3,336 (68.2)	1,990 (88.1)	5,424 (77.3)
Provider-initiated preterm births (piPTB)^a^	3,404 (24.0)	1,550 (31.7)	266 (11.8)	1,588 (22.6)
Preterm birth, labour status unknown^a^	12 (0.1)	7 (0.1)	4 (0.2)	1 (0.0)
Medically indicated piPTB^b^	2,920 (85.8)	1,412 (91.1)	236 (88.7)	1,272 (80.1)
Not medically indicated piPTB^b^	333 (9.8)	79 (5.1)	7 (2.6)	247 (15.6)
Indication unknown, piPTB^b^	151 (4.4)	59 (3.8)	23 (8.6)	69 (4.3)

^a^Denominator is all preterm births.

^b^Denominator is all provider-initiated preterm births.

Table 2 shows maternal and neonatal characteristics and preterm birth risk factors across all included pregnancies. Women experiencing spPTB trended significantly towards younger age, less education, less antenatal care, lower maternal height, as well as higher rates of malaria (2.1% vs 1.1%, p < 0.001) and pre-eclampsia (3.5% vs 2.6%, p < 0.001) compared to women with term deliveries (Table 2). Vaginal deliveries (79.7% vs 72.0%, p < 0.001) were more common among spPTB. Differences between piPTB and term deliveries were similar, however rates of malaria were slightly lower (0.5% vs 1.1%, p = 0.003), while pyelonephritis/UTI (8.2% vs 7.1%, p = 0.013), diabetes (2.9% vs 0.8%, p < 0.001) and pre-eclampsia (18.2% vs 2.6%, p < 0.001) were higher in women with piPTB. Caesarean section (66.4% vs 28.0%, p < 0.001) was significantly more common in piPTB. Most (97%) of all deliveries occurred in urban or peri-urban facilities, but piPTBs were more commonly in tertiary facilities (64.6%) compared to spPTB (59.1%) and term deliveries (54.7%).

**Table 2:** Global maternal and neonatal characteristics for spontaneous, provider-initiated preterm births and term births in liveborn singletons (N = 172,449)*

	**Spontaneous preterm (spPTB) N (%)**	**Chi-square p value**^**a**^	**Provider-initiated preterm birth (piPTB) N (%)**	**Chi-square p value**^**b**^	**Term N (%)**
**Total**	**10,750**		**3,404**		**158,295**
**Maternal age**		<0.001		<0.001	
<18	679 (6.3)		111 (3.3)		6,677 (4.2)
18-35	9,295 (86.5)		2,820 (82.8)		139,333 (88.0)
>35	771 (7.2)		473 (13.9)		12,233 (7.7)
Missing	5 (0.0)		0 (0.0)		52 (0.0)
**Years of education**		<0.001		0.107	
0	1,168 (10.9)		191 (5.6)		8,684 (5.5)
1 to 4	764 (7.1)		163 (4.8)		8,457 (5.3)
5 to 9	4,419 (41.1)		1,252 (36.8)		60,726 (38.4)
> = 10	4,139 (38.5)		1,731 (50.9)		77,824 (49.2)
Missing	260 (2.4)		67 (2.0)		2,604 (1.6)
**Married**	9,425 (87.7)	<0.001	3,053 (89.7)	0.708	141,701 (89.5)
Missing	16 (0.1)		5 (0.1)		186 (0.1)
**Parity**		<0.001		0.517	
Parity 0	4,950 (46.0)		1,476 (43.4)		70,192 (44.3)
Parity 1-2	4,421 (41.1)		1,530 (44.9)		6,999 (44.2)
Parity > = 3	1,352 (12.6)		394 (11.6)		17,912 (11.3)
Missing	27 (0.3)		4 (0.1)		192 (0.1)
**Number of antenatal care visits**		<0.001		<0.001	
0	1,023 (9.5)		226 (6.6)		6,425 (4.1)
1 to 3	3,691 (34.3)		717 (21.1)		33,780 (21.3)
4 or more	5,804 (54.0)		2,396 (70.4)		115,685 (73.1)
Missing	232 (2.2)		65 (1.9)		2,405 (1.5)
**Maternal height**		<0.001		<0.001	
<145 cm	446 (4.1)		141 (4.1)		4,355 (2.8)
145 – 149.9 cm	991 (9.2)		332 (9.8)		12,583 (7.9)
150 – 154.9 cm	3,375 (31.4)		967 (28.4)		42,497 (26.8)
> = 155 cm	5,299 (49.3)		1,798 (52.8)		91,722 (58.0)
Missing	639 (5.9)		166 (4.9)		7,088 (4.5)
**Antenatal medical and obstetric conditions**					
Malaria	225 (2.1)	<0.001	18 (0.5)	0.003	1,669 (1.1)
HIV	79 (0.7)	0.182	31 (0.9)	0.042	995 (0.6)
Pyelonephritis/UTI	712 (6.6)	0.065	279 (8.2)	0.013	11,228 (7.1)
Diabetes	91 (0.8)	0.442	100 (2.9)	<0.001	1,233 (0.8)
Pre-eclampsia	380 (3.5)	<0.001	619 (18.2)	<0.001	4,047 (2.6)
**Mode of delivery**		<0.001		<0.001	
Vaginal delivery	8,571 (79.7)		1,144 (33.6)		114,010 (72.0)
Caesarean section	2,178 (20.3)		2,260 (66.4)		44,281 (28.0)
Missing	1 (0.0)		0 (0.0)		4 (0.0)
**Neonates**					
Mean birth weight (SD)	2436.1 (630.5)	<0.001^c^	2308.2 (659.8)	<0.001	3154.4 (476.2)
Mean GA (SD)	34.4 (2.3)	<0.001^c^	34.2 (2.4)	<0.001	39.1 (1.2)
Female sex	5,181 (48.2)	0.240	1,623 (47.7)	0.197	77,225 (48.8)
**Facility characteristics:**					
**Location**					
Urban	9,746 (90.7)	<0.001	3,269 (96.0)	<0.001	14,887 (94.0)
Peri-urban	731 (6.8)		66 (1.9)		4,891 (3.1)
Rural	273 (2.5)		69 (2.0)		4,547 (2.9)
**Level of facility**					
Primary	347 (3.2)	<0.001	19 (0.6)	<0.001	3,471 (2.2)
Secondary	3,261 (30.3)		938 (27.6)		56,444 (35.7)
Tertiary	6,355 (59.1)		2,199 (64.6)		86,577 (54.7)
Other referral level	787 (7.3)		248 (7.3)		11,803 (7.5)

^*^12 preterm deliveries with unknown labour status excluded from this table.

^a^P value calculated by comparison between spontaneous preterm birth and term births only.

^b^P value calculated by comparison between provider-initiated preterm birth and term births only.

^c^Students t-test used to calculated p-values for continuous variables birthweight and gestational age.

At the country level (Table 3), the median singleton preterm birth rate was 8.3% (interquartile range 6.7% to 9.7%). The median proportion of preterm deliveries due to piPTB was 18.3% (interquartile range 12.6% to 34.7%). The proportion of piPTB deliveries that did not have a medical indication was generally less than 10% of all piPTBs, however facilities in Sri Lanka (23.8%), China (22.3%) and India (16.2%) had the highest proportion of non-medically indicated piPTBs. Height <145 cm ranged from 0.2% (Algeria) to 9.7% (Nepal). Malaria and HIV/AIDS were largely confined to African countries, with malaria ranging from 0.1% (Algeria) to 28.6% (Niger) and HIV/AIDS ranging from 0.2% (Algeria) to 3.7% (Uganda) in Africa. Pyelonephritis/UTI ranged from 0.0% (Cambodia) to 28.2% (Ecuador), diabetes ranged from 0.1% (Kenya, Ecuador, Nicaragua, Cambodia and Nepal) to 2.7% (Sri Lanka) and pre-eclampsia ranged from 0.4% (Sri Lanka) to 6.9% (Ecuador).

**Table 3:** Prevalence of preterm birth and maternal risk factors, by country

	**Africa**						**Latin America**								**Asia**							
	**DR Congo**	**Algeria**	**Kenya**	**Niger**	**Nigeria**	**Uganda**	**Argentina**	**Brazil**	**Cuba**	**Ecuador**	**Mexico**	**Nicaragua**	**Paraguay**	**Peru**	**Cambodia**	**China**	**India**	**Nepal**	**Philippine**	**Sri Lanka**	**Thailand**	**Vietnam**
**Number of liveborn singleton deliveries**	**3,239**	**10,493**	**2,415**	**1,225**	**1,433**	**11,739**	**4,916**	**1,890**	**10,149**	**8,481**	**18,316**	**5,002**	**2,624**	**12,803**	**5,064**	**8,740**	**17,199**	**7,440**	**8,971**	**14,037**	**8,926**	**7,439**
**Number of facilities**	**4**	**10**	**2**	**2**	**2**	**7**	**5**	**2**	**9**	**5**	**16**	**4**	**4**	**9**	**4**	**9**	**13**	**6**	**8**	**12**	**9**	**3**
	**N (%)**	**N (%)**	**N (%)**	**N (%)**	**N (%)**	**N (%)**	**N (%)**	**N (%)**	**N (%)**	**N (%)**	**N (%)**	**N (%)**	**N (%)**	**N (%)**	**N (%)**	**N (%)**	**N (%)**	**N (%)**	**N (%)**	**N (%)**	**N (%)**	**N (%)**
Term births (> = 37 weeks)^a^	2,829 (87.3)	10,013 (95.4)	2,253 (93.3)	1,110 (90.6)	1,312 (91.6)	10,767 (91.7)	4,432 (90.2)	1,689 (89.4)	9,650 (95.1)	7,895 (93.1)	16,857 (92.0)	4,669 (93.3)	2,367 (90.2)	11,729 (91.6)	4,733 (93.5)	8,247 (94.4)	14,554 (85.0)	6,764 (90.9)	8,230 (91.7)	13,069 (93.1)	7,977 (89.6)	7,129 (95.8)
All preterm births (<37 weeks)^a^	410 (12.7)	480 (4.6)	162 (6.7)	115 (9.4)	121 (8.4)	972 (8.3)	484 (9.8)	201 (10.6)	499 (4.9)	586 (6.9)	1,459 (8.0)	333 (6.7)	257 (9.8)	1,074 (8.4)	331 (6.5)	493 (5.6)	2,565 (15.0)	676 (9.1)	741 (8.3)	968 (6.9)	929 (10.4)	310 (4.2)
Spontaneous preterm birth^b^	378 (92.2)	374 (77.9)	153 (94.4)	100 (87.0)	109 (90.1)	876 (90.1)	294 (60.7)	162 (80.6)	257 (51.5)	522 (89.1)	1,019 (69.8)	239 (71.8)	161 (62.6)	682 (63.5)	281 (84.9)	314 (63.7)	2,091 (81.5)	592 (87.6)	642 (86.6)	481 (49.7)	760 (81.8)	263 (84.8)
Provider-initiated preterm birth^b^	32 (7.8)	106 (22.1)	9 (5.6)	15 (13.0)	12 (9.9)	92 (9.5)	187 (38.6)	39 (19.4)	242 (48.5)	64 (10.9)	439 (30.1)	94 (28.2)	93 (36.2)	392 (36.5)	50 (15.1)	179 (36.3)	474 (18.5)	84 (12.4)	99 (13.4)	487 (50.3)	168 (18.1)	47 (15.2)
Preterm birth, labour status unknown^b^	0 (0.0)	0 (0.0)	0 (0.0)	0 (0.0)	0 (0.0)	4 (0.4)	3 (0.6)	0 (0.0)	0 (0.0)	0 (0.0)	1 (0.0)	0 (0.0)	3 (1.2)	0 (0.0)	0 (0.0)	0 (0.0)	0 (0.0)	0 (0.0)	0 (0.0)	0 (0.0)	1 (0.1)	0 (0.0)
Medical indicated piPTB^c^	31 (96.9)	100 (94.3)	8 (88.9)	13 (86.7)	12 (100.0)	72 (78.3)	180 (96.3)	38 (97.4)	236 (97.5)	49 (76.6)	357 (81.3)	83 (88.3)	84 (90.3)	385 (98.2)	49 (98.0)	131 (73.2)	390 (82.3)	82 (97.6)	90 (90.9)	334 (68.6)	151 (89.9)	45 (95.7)
Not medically indicated piPTB^c^	1 (3.1)	1 (1.0)	0 (0.0)	1 (6.7)	0 (0.0)	4 (4.3)	6 (3.2)	1 (2.6)	0 (0.0)	7 (10.9)	48 (10.9)	11 (11.7)	4 (4.3)	2 (0.5)	1 (2.0)	40 (22.3)	77 (16.2)	0 (0.0)	0 (0.0)	116 (23.8)	11 (6.5)	2 (4.3)
Indication unknown, piPTB^c^	1 (3.1)	5 (4.7)	1 (11.1)	1 (6.7)	0 (0.0)	16 (17.4)	1 (0.5)	0 (0.0)	6 (2.5)	8 (12.5)	34 (7.7)	0 (0.0)	5 (5.4)	5 (1.3)	0 (0.0)	8 (4.5)	7 (1.5)	2 (2.4)	9 (9.1)	37 (7.6)	6 (3.6)	0 (0.0)
**Maternal morbidities**																						
Maternal height^a^																						
<145 cm	22 (0.7)	17 (0.2)	24 (1.0)	5 (0.4)	120 (8.4)	154 (1.3)	48 (1.0)	15 (0.8)	110 (1.1)	355 (4.2)	401 (2.2)	202 (4.0)	31 (1.2)	491 (3.8)	128 (2.5)	35 (0.4)	721 (4.2)	722 (9.7)	384 (4.3)	772 (5.5)	129 (1.4)	56 (0.8)
145 - 149.9 cm	88 (2.7)	53 (0.5)	89 (3.7)	11 (0.9)	46 (3.2)	503 (4.3)	219 (4.5)	52 (2.8)	448 (4.4)	949 (11.2)	1,327 (7.2)	635 (12.7)	81 (3.1)	1,505 (11.8)	527 (10.4)	122 (1.4)	1,842 (10.8)	1,575 (21.2)	742 (8.3)	2,024 (14.4)	727 (8.1)	341 (4.6)
150 - 154.9 cm	407 (12.6)	693 (6.6)	271 (11.2)	56 (4.6)	255 (17.8)	1,832 (15.6)	848 (17.2)	218 (11.5)	1,686 (16.6)	2,548 (30.0)	4,867 (26.6)	1,769 (35.4)	424 (16.2)	3,776 (29.5)	1,678 (33.1)	1,171 (13.4)	8,667 (50.6)	3,801 (51.1)	2,719 (30.3)	4,569 (32.5)	2,274 (25.5)	2,313 (31.1)
> = 155 cm	2,669 (82.4)	9,686 (92.3)	1,209 (50.1)	1,153 (94.1)	805 (56.2)	8,271 (70.5)	3,141 (63.9)	758 (40.1)	7,905 (77.9)	4,152 (49.0)	10,612 (57.9)	2,396 (47.9)	1,876 (71.5)	6,099 (47.6)	2,644 (52.2)	7,403 (84.7)	5,848 (34.2)	1,334 (17.9)	3,935 (43.9)	6,589 (46.9)	5,663 (63.4)	4,728 (63.6)
Missing	53 (1.6)	44 (0.4)	822 (34.0)	0 (0.0)	207 (14.4)	979 (8.3)	660 (13.4)	847 (44.8)	0 (0.0)	477 (5.6)	1,109 (6.1)	0 (0.0)	212 (8.1)	932 (7.3)	87 (1.7)	9 (0.1)	41 (0.2)	8 (0.1)	1,191 (13.3)	83 (0.6)	133 (1.5)	1 (0.0)
Malaria^a^	413 (12.8)	13 (0.1)	10 (0.4)	350 (28.6)	207 (14.5)	854 (7.3)	0 (0.0)	0 (0.0)	4 (0.0)	0 (0.0)	10 (0.1)	0 (0.0)	2 (0.1)	2 (0.0)	2 (0.0)	0 (0.0)	37 (0.2)	2 (0.0)	1 (0.0)	2 (0.0)	3 (0.0)	1 (0.0)
HIV/AIDS^a^	43 (1.3)	19 (0.2)	16 (0.7)	3 (0.2)	12 (0.8)	415 (3.6)	39 (0.8)	21 (1.1)	49 (0.5)	28 (0.3)	80 (0.4)	20 (0.4)	3 (0.1)	36 (0.3)	42 (0.8)	17 (0.2)	57 (0.3)	7 (0.1)	19 (0.2)	11 (0.1)	111 (1.2)	57 (0.8)
Pyelonephritis/UTI^a^	234 (7.2)	267 (2.5)	65 (2.7)	34 (2.8)	1 (0.1)	912 (7.8)	216 (4.4)	243 (12.9)	487 (4.8)	2,390 (28.2)	3,715 (20.3)	266 (5.3)	53 (2.0)	2,602 (20.3)	2 (0.0)	6 (0.1)	84 (0.5)	5 (0.1)	499 (5.6)	86 (0.6)	15 (0.2)	37 (0.5)
Diabetes^a^	12 (0.4)	103 (1.0)	3 (0.1)	3 (0.2)	3 (0.2)	30 (0.3)	66 (1.3)	39 (2.1)	213 (2.1)	11 (0.1)	129 (0.7)	6 (0.1)	12 (0.5)	21 (0.2)	5 (0.1)	70 (0.8)	45 (0.3)	6 (0.1)	42 (0.5)	385 (2.7)	199 (2.2)	14 (0.2)
Pre-eclampsia^a^	34 (1.0)	67 (0.6)	15 (0.6)	14 (1.1)	16 (1.1)	371 (3.2)	114 (2.3)	77 (4.1)	210 (2.1)	584 (6.9)	867 (4.7)	135 (2.7)	87 (3.4)	830 (6.5)	108 (2.1)	184 (2.1)	567 (3.3)	88 (1.2)	363 (4.0)	54 (0.4)	188 (2.1)	75 (1.0)

^a^Percentage calculated using number of liveborn singleton deliveries in country X as denominator.

^b^Percentage calculated using number of all preterm births in country X as denominator.

^c^Percentage calculated using number of all provider-initiated preterm births in country X as denominator.

Table 4 shows the bivariate and multivariate risk factor analysis for preterm birth by regions and globally. In the pooled global analysis, all maternal morbidities (maternal height <145 cm, 145-149.9 cm and 150-154.9 cm, diabetes, pre-eclampsia, UTI/pyelonephritis) were significantly associated with spPTB, however these associations varied in magnitude and significance at the regional level. Diabetes was significant in Latin America and Africa and pre-eclampsia was significant in Africa and Asia. Maternal height <145 cm and 145-149.9 cm, diabetes and pre-eclampsia were significantly associated with piPTB. In Africa, malaria was significantly associated with spPTB (AOR 1.67, 95% CI 1.32 – 2.11) while HIV/AIDS did not reach significance for spPTB (AOR 1.17, 95% CI 0.79 – 1.73) or piPTB (AOR 1.09, 95% CI 0.86 – 1.40).

**Table 4:** Crude and adjusted odds of preterm birth (spontaneous and provider-initiated) associated with maternal morbidities, by region and globally

		**Spontaneous preterm birth (spPTB)**		**Provider-initiated preterm birth (piPTB)**	
		**OR (95% CI)**	**AOR (95% CI)**	**OR (95% CI)**	**AOR (95% CI)**
**Latin America**	Maternal height				
	<145 cm	1.15 (0.92 – 1.42)	1.05 (0.85 – 1.28)	0.99 (0.72 – 1.36)	1.15 (0.85 – 1.57)
	145 – 149.9 cm	1.08 (0.95 – 1.23)	1.06 (0.93 – 1.20)	0.92 (0.76 – 1.11)	0.99 (0.82 – 1.20)
	150 – 154.9 cm	1.10 (1.01 – 1.19)	1.06 (0.96 – 1.17)	0.93 (0.82 – 1.05)	0.99 (0.86 – 1.13)
	> = 155 cm	Ref	Ref	Ref	Ref
	Pyelonephritis/UTI	1.07 (0.97 – 1.18)	1.20 (1.01 – 1.42)*	1.08 (0.94 – 1.23)	1.27 (0.96 – 1.67)
	Diabetes	1.34 (0.93 – 1.92)	1.72 (1.15 – 2.58)*	3.47 (2.48 – 4.85)	2.23 (1.48 – 3.38)*
	Pre-eclampsia	1.38 (1.18 – 1.62)	1.18 (0.92 – 1.53)	7.80 (6.90 – 8.83)	7.43 (5.69 – 9.69)
**Africa**	Maternal height				
	<145 cm	2.02 (1.44 – 2.83)	1.46 (0.81 – 2.62)	1.91 (0.78 – 4.67)	1.21 (0.75 – 1.96)
	145 – 149.9 cm	1.22 (0.93 – 1.62)	0.91 (0.72 – 1.16)	1.73 (0.94 – 3.19)	1.13 (0.80 – 1.58)
	150 – 154.9 cm	1.51 (1.33 – 1.72)	1.22 (1.04 – 1.43)*	1.33 (0.93 – 1.89)	1.05 (0.92 – 1.19)
	> = 155 cm	Ref	Ref	Ref	Ref
	*Malaria*	2.00 (1.72 – 2.33)	1.67 (1.32 – 2.11)*	1.06 (0.64 – 1.76)	1.01 (0.84 – 1.21)
	*HIV/AIDS*	1.10 (0.78 – 1.54)	1.17 (0.79 – 1.73)	1.61 (0.75 – 3.42)	1.09 (0.86 – 1.40)
	Pyelonephritis/UTI	0.91 (0.73 – 1.13)	0.91 (0.66 – 1.24)	0.44 (0.20 – 0.99)	0.91 (0.81 – 1.02)
	Diabetes	1.76 (1.06 – 2.92)	1.96 (1.03 – 3.71)*	4.71 (2.06 – 10.75)	1.66 (1.09 – 2.55)*
	Pre-eclampsia	1.40 (1.02 – 1.93)	1.55 (1.11 – 2.17)*	13.57 (9.74 – 18.91)	3.59 (2.02 – 6.40)*
**Asia**	Maternal height				
	<145 cm	2.12 (1.88 – 2.41)	1.39 (1.13 – 1.71)*	1.94 (1.56 – 2.41)	1.58 (1.26 – 1.98)*
	145 – 149.9 cm	1.60 (1.46 – 1.76)	1.21 (1.08 – 1.36)*	1.48 (1.26 – 1.73)	1.25 (1.02 – 1.53)*
	150 – 154.9 cm	1.54 (1.45 – 1.64)	1.13 (1.01 – 1.26)*	1.18 (1.05 – 1.32)	1.11 (0.97 – 1.27)
	> = 155 cm	Ref	Ref	Ref	Ref
	Pyelonephritis/UTI	1.53 (1.21 – 1.95)	1.17 (0.78 – 1.77)	1.25 (0.78 – 2.01)	1.30 (0.68 – 2.49)
	Diabetes	0.82 (0.60 – 1.12)	1.15 (0.82 – 1.62)	3.83 (2.91 – 5.06)	2.58 (1.59 – 4.19)*
	Pre-eclampsia	1.64 (1.39 – 1.94)	1.26 (1.00 – 1.59)*	7.69 (6.56 – 9.02)	8.34 (6.54 – 10.64)*
**Global**	Maternal height				
	<145 cm	1.77 (1.60 – 1.96)	1.30 (1.10 – 1.52)*	1.65 (1.39 – 1.97)	1.47 (1.23 – 1.77)*
	145 – 149.9 cm	1.36 (1.27 – 1.46)	1.15 (1.06 – 1.24)*	1.35 (1.20 – 1.52)	1.18 (1.02 – 1.36)*
	150 – 154.9 cm	1.38 (1.32 – 1.44)	1.11 (1.04 – 1.19)*	1.16 (1.07 – 1.26)	1.08 (0.98 – 1.19)
	> = 155 cm	Ref	Ref	Ref	Ref
	Pyelonephritis/UTI	0.93 (0.86 – 1.01)	1.16 (1.01 – 1.33)*	1.17 (1.03 – 1.32)	1.24 (0.96 – 1.59)
	Diabetes	1.09 (0.88 – 1.35)	1.41 (1.09 – 1.82)*	3.86 (3.14 – 4.74)	2.51 (1.81 – 3.47)*
	Pre-eclampsia	1.40 (1.26 – 1.55)	1.25 (1.05 – 1.49)*	8.47 (7.72 – 9.29)	8.17 (6.80 – 9.83)*

*adjusted odds ratios that reached statistical significance (95% CI excludes 1).

**Figure 6:**
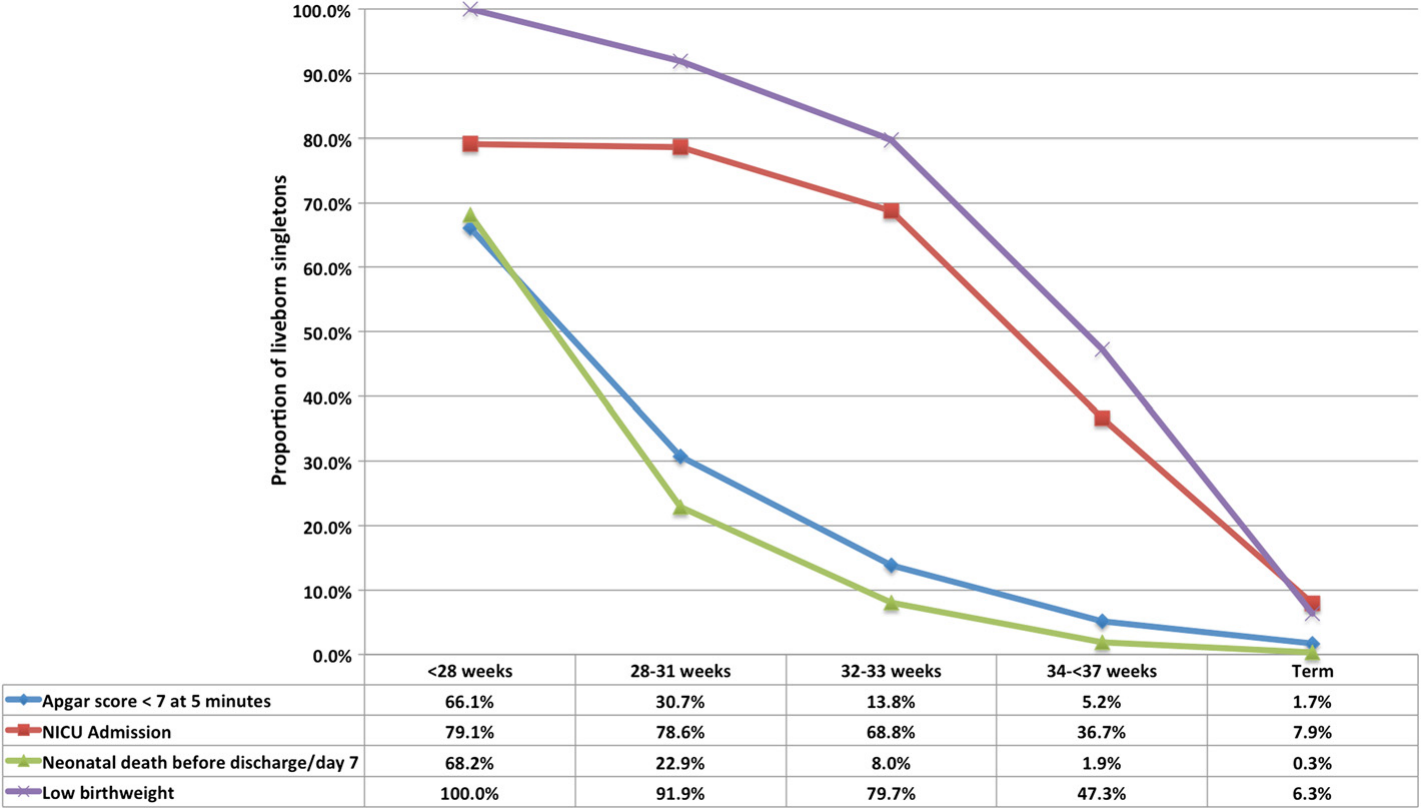
Perinatal outcomes in spontaneous preterm births.

**Figure 7:**
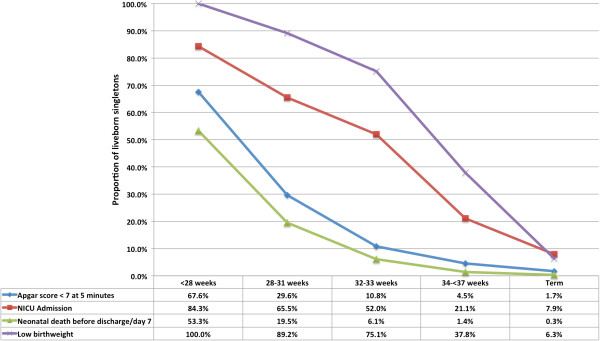
Perinatal outcomes in provider-initiated preterm birth.

For both spPTB and piPTB groups, the prevalence of all adverse neonatal outcomes decreased with increasing gestational age (Figures 6 and 7). Rates of adverse outcomes by gestational age bands were comparable between spPTB and piPTB neonates, however the rates of early neonatal death before discharge/day 7 were consistently higher in piPTB neonates.

## Discussion

We used a dataset of 172,461 deliveries across 145 facilities in 22 LMICs to examine the contribution of common maternal conditions to the risk of spPTB and piPTB independently in LMICs, as well as the associated perinatal outcomes. The accepted figure for provider-initiated preterm deliveries is 15-20%, [11] however we found a piPTB rate of 24.0%, likely reflecting the higher rate of intervention in our facility-based sampling frame. In sub-Saharan African countries where resources for obstetric intervention are often very limited [24] the non-medically indicated piPTB rate was lower (2.6%). The Latin American piPTB rate (31.7%) is likely due to lower CS thresholds for and wider availability of elective CS in these countries [25], however the rate of elective piPTB deliveries in this region was also quite low (5.1%).

The most surprising finding was the non-medically indicated piPTB rate in Asia accounting for over 15% of all provider-initiated preterm deliveries, driven by facilities in Sri Lanka, China and India. While the reason for this is unclear, it could be due to gestational age estimation errors, mis-documentation of indications for delivery, maternal demand for elective deliveries (despite being preterm) or a failure by providers to recognize the risks of elective preterm deliveries. Elective deliveries should only be performed when there is a high degree of confidence in the gestational age estimate as they increase the risk of some adverse maternal outcomes [26-28], and we have shown that rates of neonatal morbidity and mortality at 34- < 37 weeks were considerably higher. In resource-constrained settings, reducing these rates also allows resource redirection to mothers in medical need of intervention. Maternal characteristics associated with preterm deliveries were consistent with the literature [10,13] however in several countries and morbidities, the extremely low prevalence is suggestive of significant under-diagnosis and/or a lack of universal screening protocols. There is a paucity of large-scale maternal morbidity prevalence data from LMICs, particularly for UTI’s, pre-gestational diabetes and pre-eclampsia - our data, while suggestive of consistent under-recognition of common morbidities, are the largest and best available for several LMICs.

Others have reported on the increased risk of PTB with low maternal height, ranging from OR 1.17 to 1.61 [29-31], however we have shown this occurs independently for spPTB and piPTB at <145 cm and 145-149.9 cm. The association with spPTB was stronger in women in Asia where the prevalence of maternal height <145 cm was higher, while Latin America and Africa did not reach significance – this could be due to the lower prevalence of shorter women in these regions, malnutrition increasing the risk of preterm birth [32] or potentially a racial predisposition at play. The relationship with piPTB may be due to the clinician’s desire to avoid obstructed labor associated with cephalopelvic disproportion [33].

Malaria can contribute to almost 25% of maternal deaths in endemic settings [34] and intermittent treatment during pregnancy has been shown to reduce preterm birth [35]. Malaria has been associated with 2-3 times the risk of preterm birth [36-39], supported by the 67% increase in odds in our African data. With regards to HIV, a meta-analysis of 31 studies by Brocklehurst and colleagues reported OR 1.83 (1.63 – 2.06) for preterm delivery associated with HIV infection in pregnancy [40] while Lopez and colleagues demonstrated increased odds for spPTB (AOR 2.1, 1.5 – 3.0) and piPTB (AOR 3.2, 1.8 - 5.7) [41]. Our failure to find an association is likely related to the HIV rates in our dataset being considerably lower than national data. This may be due to under-detection, under-recognition or under-documentation of HIV infection during delivery. Furthermore, universal HIV screening may not be in use in these facilities, or mothers are declining testing or not reporting. Regardless, under-diagnosis is a cause for concern in the larger African facilities that participated in the WHOGS, where obstetric services and HIV diagnostic capacity are likely higher than average.

The increased risk of prematurity in mothers with pre-gestational diabetes is nearly four-fold higher [42], however this is at least partly driven by iatrogenic prematurity [43] - whether pre-gestational diabetes is an independent risk factor for spPTB is unclear. Our findings support the 60% increase in the odds of spPTB reported by Sibai and colleagues [44]. Given the growing global epidemic of diabetes and obesity, preterm birth rates will likely be driven higher by this relationship. Our findings also support studies that have implicated urinary tract infections [45] and pre-eclampsia [46,47] in preterm birth. Pre-eclampsia is a leading indication for piPTB [46] - the lower magnitude risk of piPTB in African countries (AOR 3.59) compared to overall (AOR 8.17) likely reflects a relatively lower number of women with pre-eclampsia being managed with caesarean, or perhaps comparative overuse in other settings. It is important to note that many of these conditions can co-occur in LMICs, and improved access and use of health prevention strategies (such as antenatal screening programmes) in these settings will likely yield a secondary benefit of reduced spPTB rates. Although most adverse perinatal outcomes decreased in prevalence with increasing gestational age, the NICU admission rate was generally lower than we expected, (particularly for early preterm infants) possibly due to poor NICU access. In an estimation exercise for CHERG, Blencowe et al estimated that in countries with <80% national facility delivery rates, <50% of infants had access to a neonatal intensive care unit (personal communication). While piPTB may save an infant’s life, the higher early neonatal mortality rate following piPTB likely reflects the maternal indication.

Our analysis had several strengths - it was conducted in a large, multi-country dataset and stratified by type of preterm birth and region. Most preterm analyses are from high-income settings and are generally not segregated by preterm phenotypes; they are unable to distinguish increased risks of of spPTB from an increased propensity of providers to intervene (such as for maternal height <145 cm, diabetes and pre-eclampsia). These epidemiological data on preterm birth groups and maternal morbidities are the best available for several LMICs. Despite our efforts controlling for multiple confounders, some limitations persist. The sample was primarily drawn from larger, urban facilities and therefore subject to reporting and/or selection biases (higher rates of complicated pregnancies, or wealthier and more urban mothers attending tertiary care level facilities). Higher CS rates in some countries and facilities reflect this, therefore these findings are not representative of the population and can only be extrapolated to similar settings. Furthermore, the data is based on retrospective review of medical records (which may have been sub-optimal) and morbidity surveillance may not have been universal in these facilities. The method of diagnosis, timing, severity and treatment of these morbidities, as well as the method of gestational age determination, was not recorded. We lacked data on some relevant PTB risk factors, such as smoking, birth spacing, sexually transmitted infections, physical exertion and lifestyle risk factors. Intrauterine fetal deaths were not included. It is important to acknowledge that women who undergo preterm birth and/or provider interventions may be more likely to have a risk factor diagnosed than other women, potentially distorting associations.

## Conclusion

In facility deliveries in 22 low- and middle-income countries, 24.0% of preterm births were provider-initiated, yet nearly 10% of these were without a medical indication and potentially avoidable. Maternal height <155 cm, pyelonephritis/UTI, diabetes, pre-eclampsia and malaria independently increased the odds of spontaneous preterm birth. Furthermore, malaria was significantly associated with spontaneous preterm birth in Africa. Aside from maternal height, these common maternal conditions are amenable to screening, prevention and treatment – failure to do so increases the risk of preterm birth and associated neonatal morbidity and mortality in LMICs. Strategies to reduce preterm birth in LMICs must prioritise maternal antenatal screening and treatment of these conditions.

## Abbreviations

AOR: Adjusted odds ratio; CHERG: Child health epidemiology reference group; CS: Caesarean section; ICU: Intensive care unit; HIV/AIDS: Human immunodeficiency virus/acquired immunodeficiency syndrome; LMICs: Low- and middle-income countries; NICU: Neonatal intensive care unit; OECD: Organization for economic cooperation and development; OR: Odds ratio; piPTB: Provider-initiated preterm birth; PTB: Preterm birth; spPTB: Spontaneous preterm birth; USAID: United States Agency for International Development; UTI: Urinary tract infection; WHO: World Health Organization; WHOGS: World Health Organization Global Survey on Maternal and Perinatal Health.

## Competing interests

The authors declare they have no competing interests.

## Authors’ contributions

JPV and ACL designed the analysis, JPV performed the analysis and JPV, ACL and JPS drafted the paper. All authors contributed to and approved the final version of the manuscript. JPV had full access to the dataset in the study and takes responsibility for the integrity of the data and the accuracy of the data analysis. The views contained within this manuscript represent the views of the named authors alone.

## Pre-publication history

The pre-publication history for this paper can be accessed here:

http://www.biomedcentral.com/1471-2393/14/56/prepub
